# Feasibility, acceptability, and preliminary efficacy of an LGBTQ+ inclusive communication skills training for multidisciplinary oncology healthcare providers^☆^

**DOI:** 10.1016/j.pecinn.2026.100457

**Published:** 2026-02-01

**Authors:** William E. Rosa, Smita C. Banerjee, Meghan McDarby, Amanda Kastrinos, Elizabeth Schofield, Kimberly D. Acquaviva, Koshy Alexander, Patricia A. Parker

**Affiliations:** a Department of Psychiatry and Behavioral Sciences, Memorial Sloan Kettering Cancer Center, New York, NY, USA; b Department of Public Health Sciences, Medical University of South Carolina, Charleston, SC, USA; c School of Nursing, University of Virginia, Charlottesville, VA, USA; d Department of Medicine (Geriatrics), Memorial Sloan Kettering Cancer Center, New York, NY, USA

**Keywords:** Experiential learning, Health communication, LGBT, LGBTQ+, Multidisciplinary, Oncology, Role play, Sexual and gender minorities, SGM

## Abstract

**Objective:**

To test the feasibility, acceptability, and preliminary efficacy of an LGBTQ+ inclusive communication skills training program.

**Methods:**

Two modules focused on basic LGBTQ+ sensitivity and communication with LGBTQ+ patients' families were delivered for multidisciplinary oncology healthcare professionals (HCPs) during two training sessions. Feasibility was determined through recruitment rate and adherence. Acceptability was evaluated based on course evaluations. Pre−/post-training assessments included LGBTQ+ healthcare knowledge, beliefs towards LGBTQ+ people, and self-efficacy.

**Results:**

Participants were predominantly female (83%) and included 1 nurse, 2 chaplains, 8 nurse practitioners, and 7 physicians. Of the 22 HCPs who initially expressed interest and registered for the training, a total of 18 (82%) participated. 89% of participants completed post-training evaluations within one week. The training was rated favorably; 80% of participants “agreed” or “strongly agreed” with 13 of 15 course evaluation items. From pre- to post-training, LGBTQ+ health knowledge increased for 6 of 14 items and self-efficacy improved significantly for all 9 items evaluated (total score, d = 1.32); beliefs did not change between assessment points.

**Conclusions:**

An LGBTQ+ communication training for multidisciplinary oncology HCPs was feasible to deliver, acceptable to participants, and led to increases in LGBTQ+ healthcare knowledge and participant self-efficacy from pre- to post-training.

**Innovation:**

LGBTQ+ inclusive communication training fills a crucial gap in HCP research, education, and practice, providing a novel experiential learning approach to mitigating health inequities for LGBTQ+ patients and their families.

## Highlights

• LGBTQ+ people with cancer and other serious illnesses experience pervasive discriminatory care
• Healthcare professionals (HCPs) often lack LGBTQ+ health knowledge and feel ill equipped to communicate respectfully
• HCPs should be trained to communicate empathically with both LGBTQ+ patients and their family caregivers
• LGBTQ+ inclusive communication training for oncology HCPs can bolster relevant knowledge and self-efficacy
• Communication training to improve care for LGBTQ+ people fills crucial HCP knowledge, education, and practice gaps

## 1 Introduction

LGBTQ+ people (i.e., lesbian, gay, bisexual, transgender, queer/questioning) with serious illnesses, and their family members, consistently report discriminatory experiences, including disrespectful communication by healthcare professionals (HCPs) [1], [2], [3], [4], [5], [6], [7], [8], [9], [10], [11]. In turn, HCPs report being underprepared to provide LGBTQ+ inclusive care (e.g., discomfort with eliciting sexual orientation and gender identity (SOGI) data, inadequate LGBTQ+ health knowledge) [12], [13], [14], [15]. These communication and interpersonal factors, in conjunction with LGBTQ+ patients' potential fear and worry about mistreatment, lower insurance coverage, and dissatisfaction with cancer care, may result in delayed or avoided healthcare encounters or decisions to stop treatment [1], [16], [17].

Despite myriad initiatives to improve care for LGBTQ+ people with cancer [18], [19], [20], [21], [22], [23], training opportunities to bolster empathic, effective, and efficient communication for LGBTQ+ patients and their families are lacking. To address the negative healthcare experiences of this population, as well as HCP knowledge and education gaps regarding sensitive and respectful LGBTQ+ care, we developed two LGBTQ+ inclusive communication skills training modules (i.e., LGBTQ+ 101 and LGBTQ+ 102) for multidisciplinary oncology HCPs [24], [25]. The purpose of this study was to evaluate learner outcomes of these two training modules delivered in an integrated program format to assess their feasibility, acceptability, and preliminary efficacy.

## 2 Methods

### 2.1 Design

This was a single-site feasibility, acceptability, and preliminary efficacy study of two LGBTQ+ inclusive communication skill modules (i.e., LGBTQ+ 101 and 102) for multidisciplinary HCPs. Module content and format are described in section 2.4.

### 2.2 Sample and setting

Participants included multidisciplinary HCPs (e.g., chaplains, nurses, nurse practitioners, physicians, social workers, clinical fellows) working in our comprehensive cancer center's Supportive Care Service and providing direct adult patient care.

### 2.3 Procedures

During August and September 2024, we sent two invitations by email to Supportive Care Service HCPs to participate in the LGBTQ+ inclusive communication training. Two trainings, one virtual and one in-person, were then delivered and evaluated by our Communication Skills Training and Research Program (Comskil) faculty in November 2024. Enrolled participants completed both LGBTQ+ 101 and 102 modules during a 3-h training, as well as pre- and post- training measures online via RedCAP survey. Both the training and reported research received exemption from our Institutional Review Board.

### 2.4 Module content and format

The content of the LGBTQ+ 101 and 102 modules has been described previously and are briefly outlined here [24], [25]. The first module (i.e., LGBTQ+ 101) focused on basic cultural sensitivity (e.g., respectful use of patient-identified pronouns), and aimed to teach participants (a) strategies to create a safe, welcoming environment; (b) skills to invite all patients to share SOGI information; and (c) tasks involved in inquiring about and inviting LGBTQ+ patients' family of choice in medical decision-making [24]. In prior testing of LGBTQ+ 101, 33 HCPs participants rated the training positively while demonstrating significant improvements in LGBTQ+ healthcare knowledge, self-efficacy, beliefs towards lesbian, gay, bisexual, and transgender persons, and LGBTQ+ sensitive language use skills following the training [24].

Building on LGBTQ+ 101, the second module focused on communication specifically for the family members of LGBTQ+ patients with cancer (i.e., LGBTQ+ 102) [25]. LGBTQ+ 102 equipped HCPs with skills needed to engage family members in shared decision-making with the patient, identify family concerns and strengths, and affirm both biological and chosen family structures. During two pilot trainings of the LGBTQ+ 102 module, participants (*n* = 19) reported favorable learning experiences and emphasized the value of this module for HCPs to improve overall care quality for LGBTQ+ people [25].

In the current study, LGBTQ+ 101 and 102 were delivered in a sequential and integrated format. Each module was comprised of didactic lectures that summarized previous literature on LGBTQ+ cancer care; the rationale for and role of inclusive communication; and recommended communication strategies with exemplary skill demonstration videos. Following an abridged version of the 101 didactic, faculty facilitated a fishbowl discussion with participants about concerns and challenges in delivering LGBTQ+ inclusive care. After the 102 didactic, participants were split into small groups for role-play sessions with Comskil facilitators. These role plays (i.e., experiential learning exercises) provided an opportunity for participants to practice strategies from the didactic with a lesbian, gay, bisexual, or transgender standardized patient (SP)/family member played by a professionally trained actor.

### 2.5 Measures

#### 2.5.1 Demographic, work-related, and other variables

Demographic data (e.g., age, race, gender) and work-related variables (e.g., profession, level of education) were collected, in addition to learners' pronouns and two questions about whether they had lesbian, gay, bisexual and/or transgender friends.

#### 2.5.2 Feasibility

Feasibility was determined through recruitment rate and adherence. A 50% recruitment rate between those who initially expressed interest and then participated in the training was considered feasible based on prior Comskil trainings [26]. Adherence was defined as the proportion of participants who completed all post-module evaluations within one week of the training (70% completion rate threshold).

#### 2.5.3 Acceptability

Acceptability was determined based on participant satisfaction per course evaluation outcomes. Participants completed a post-training evaluation developed to capture engagement, novelty, and reflectiveness using a 15-item, 5-point Likert scale (1–5, strongly disagree to strongly agree) and modeled after prior Comskil evaluation measures [26].

#### 2.5.4 LGBTQ+ healthcare knowledge

LGBTQ+ healthcare knowledge was evaluated using an adapted version of established measures [14], [27], with four additional items added by the study team. The overall measure consisted of 14 items scored as true/false/don't know. Correct answers were scored as 1 and others 0. Overall knowledge scores were calculated by summing number of correct scores.

#### 2.5.5 Beliefs towards LGBTQ+ people

Beliefs towards lesbian, gay, and bisexual people were evaluated using a shortened version of the Sexual Orientation Beliefs Scale (11 item 7-point Likert scale, strongly disagree to strongly agree) [28] and beliefs towards transgender people were assessed using a shortened version of the Genderism and Transphobia Scale (15 item 7-point Likert scale, strongly agree to strongly disagree) [29]. Higher scores on both scales indicate favorable beliefs towards lesbian, gay, bisexual, and transgender people.

#### 2.5.6 Self-efficacy

Self-efficacy was assessed based on prior measures created and implemented by the study team [26], consisting of 9 items using a 5-point Likert scale (strongly disagree to strongly agree). Higher scores indicate high self-efficacy.

### 2.6 Data analysis

Descriptive statistics were generated for demographic data, recruitment, and adherence metrics. Training evaluation was analyzed descriptively with “agree” or “strongly agree” responses indicating satisfaction and acceptability. To assess knowledge, beliefs, and self-efficacy scores between pre- and post-training, paired *t*-tests were utilized, and Cohen's d effect sizes were generated.

## 3 Results

### 3.1 Demographic, work-related, and other variables

Eight HCPs attended the virtual training (1 nurse, 1 chaplain, 6 nurse practitioners), and 10 HCPs attended the in-person training (1 chaplain, 2 nurse practitioners, 7 physicians). Of the total sample (*N* = 18), half of the participants were white (50%), 83% were female, and 39% were physician trainees (i.e., clinical fellows) (Table 1). 94% reported having lesbian, gay, and/or bisexual friends; 50% endorsed having transgender friends.

**Table 1:** Participant Baseline Characteristics (*n* = 18).

Characteristic	n (%)
Age, M (SD)	40.9 (10.6)
Race	
White	9 (50%)
Black or African American	2 (11%)
Asian	5 (28%)
Native Hawaiian or Pacific Isl.	1 (6%)
Multiracial	1 (6%)
Gender	
Man	3 (17%)
Woman	14 (78%)
Missing/Refused	1 (6%)
Pronouns	
She/her/hers	15 (83%)
He/him/his	3 (17%)
Profession	
Chaplain	2 (11%)
Nurse	1 (1%)
Nurse Practitioner	8 (44%)
Physician	7 (39%)
Education	
4-year college (BA, BS)	1 (6%)
Master's degree	7 (39%)
Doctoral degree	2 (11%)
Professional degree (JD, MD)	8 (44%)
Have lesbian/gay/bisexual friends: Yes	17 (94%)
Have transgender friends: Yes	9 (50%)

### 3.2 Feasibility

Twenty-two HCPs initially expressed interest in and registered for the training, and 82% (*n* = 18), participated. A total of 16 HCPs (89%) completed the post-training evaluation within one week; one additional participant completed the post-training evaluation 10 days following the training.

### 3.3 Acceptability

Participants rated the training favorably, with more than 80% indicating that they “agreed” or “strongly agreed” with 13 of the 15 evaluation items for the role plays (Table 2). Items for novelty were rated less favorably than those for engagement and reflectiveness.

**Table 2:** Course Evaluations (15 items).

Item	Disagree	Agree
The role play was interesting to me.	0 (0%)	16 (100%)
I got easily distracted during the role play. *	1 (6%)	15 (94%)
I enjoyed this role play.	2 (13%)	14 (88%)
This role play was boring. *	0 (0%)	16 (100%)
I've never done anything like what I did in the role play today.	13 (81%)	3 (19%)
The role play was different than other communication skills training I have participated in.	5 (31%)	11 (69%)
The role play was unique.	3 (19%)	13 (81%)
This role play made me think about the importance of communication skills.	1 (6%)	15 (94%)
This role play made me think about reasons for making changes in my communication with LGBTQ+ patients.	0 (0%)	16 (100%)
This role play made me think about specific things I can do about my communication skills.	0 (0%)	16 (100%)
This role play helped me figure out how I can incorporate communication skills in my clinical interactions regularly.	1 (6%)	15 (94%)
This role play encouraged me to maintain my communication skills.	1 (6%)	15 (94%)
The role play provided new information about communication skills and process tasks.	1 (6%)	15 (94%)
The role play made me think about my communication skills with LGBTQ+ patients.	0 (0%)	16 (100%)
The role play made me think about my peers' communication skills.	1 (6%)	15 (94%)

Note. Items with an asterisk were reverse-scored for agreement/disagreement in this table only.

### 3.4 LGBTQ+ healthcare knowledge

On average, LGBTQ+ healthcare knowledge scores increased from pre-training (*M =* 11.8, *SD =* 1.5) to post-training (*M =* 12.6, *SD =* 1.6), *t* = 1.4, *p* = 0.2. Specifically, there were knowledge increases on 6 of the items (#’s 4, 6–8, 11, 13), no change on 5 items (#’s 2, 3, 9, 10, 12), and decreases in correct scores on 3 items (#’s 1, 5, 14). Table 3 provides scores for each knowledge item, as well as overall pre- to post-training changes.

**Table 3:** LGBTQ+ Healthcare Knowledge (14 items).

Item	Pre % correct	Post % correct	Χ^2^	p
Lesbian, gay, and bisexual people avoid accessing healthcare due to difficulty communicating with HCPs.	100%	88%	0.50	0.48
Trans people avoid accessing healthcare due to difficulty communicating with HCPs.	94%	94%	0.00	>0.99
Among adolescents, there is an association between being LGBTQ+ and suicide/suicidal ideation/suicidal tendencies.	100%	100%	NA	NA
Lesbians are less likely to abuse alcohol than heterosexual women.	59%	76%	0.67	0.41
The incidence of depression in older gay and lesbian people is greater than in the non-LGBTQ+ population.	100%	93%	0.00	>0.99
Heterosexual women are more likely to be smokers than lesbian women.	35%	71%	5.00	0.025
Trans men (people born as women who identify as men) who have had a mastectomy are at risk for breast cancer.	65%	82%	3.00	0.083
Trans women (people born as male who identify as women) who have undergone gender affirming surgery are at a risk for prostate cancer.	76%	94%	3.00	0.083
HCPs should create an environment that encourages disclosure of sexual orientation and gender identity.	100%	100%	NA	NA
The Joint Commission recommends hospitals create welcoming and safe environments for LGBTQ+ patients.	100%	100%	NA	NA
Non-biological or ‘chosen family’ members that serve as decision-makers and social support are common in the LGBTQ+ population.	94%	100%	NA	NA
Clinicians should always defer to biological family members, such as parents and siblings, when an LGBTQ+ patient is unable to make their decisions at end of life.	100%	100%	NA	NA
Biological relationship takes legal precedence in surrogate decision-making over a same-sex unmarried partner who is the health proxy.	82%	88%	0.33	0.56
The partners of LGBTQ+ people are less likely to suffer discrimination and mistreatment by clinicians than the partners of non-LGBTQ+ people.	100%	88%	0.50	0.48
Knowledge Score, M (SD)	11.8 (1.5)	12.6 (1.6)	t = 1.4	0.20

Note. Although 17 participants completed each of the assessments (pre and post), only 16 completed both. Percentages and means are based on all available data; all *p*-values are based on *n* = 16. NA indicates no variability between pre-training and post-training responses for those that completed both. Standardized effect size for the increase in knowledge score is d = 0.41.

### 3.5 Beliefs

Beliefs towards lesbian, gay, and bisexual people (*M =* 1.8, *SD =* 0.5; Table 4**)** and beliefs towards transgender people (*M =* 1.3, *SD =* 0.4; Table 5) remained unchanged from pre- to post-training.

**Table 4:** Beliefs Towards Lesbian, Gay, and Bisexual People (18 items).

Beliefs towards Lesbian, Gay, and Bisexual People	Pre M (SD)	Post M (SD)	d	p-val
Sexual orientation is a category with distinct boundaries: A person is either gay/lesbian or heterosexual.	1.4 (0.5)	1.3 (0.5)	−0.26	0.33
People who identify as bisexual are confused about their true sexual orientation.	1.2 (0.4)	1.2 (0.4)	0	>0.99
Sexual orientation is a category with clear boundaries: A person is either gay/lesbian, bisexual, or heterosexual.	1.4 (0.5)	1.3 (0.5)	−0.26	0.33
It is possible to be partially or somewhat gay or straight.	2.3 (1.1)	2.9 (1.5)	0.41	0.13
A person has only one true sexual orientation.	1.5 (0.8)	1.3 (0.5)	−0.09	0.72
People may reasonably identify as two sexual orientations at the same.	2.2 (0.9)	2.1 (1.0)	−0.21	0.43
People who share the same sexual orientation pursue common goals.	1.9 (0.8)	1.5 (0.7)	−0.41	0.14
People with the same sexual orientation share a common fate.	1.6 (0.7)	1.5 (0.6)	−0.21	0.43
Individuals with the same sexual orientation seem to be connected to one another by some invisible link.	2.1 (1.2)	1.8 (1.2)	−0.32	0.22
People who have the same sexual orientation are very similar to one another.	1.5 (0.7)	1.7 (1.1)	0.22	0.39
There are more similarities than differences among people who have the same sexual orientation.	1.8 (1.0)	1.4 (0.7)	−0.61	0.03
Individuals choose their sexual orientation.	1.5 (1.1)	1.6 (1.1)	0.13	0.61
People have control over changing or keeping their sexual orientation.	1.6 (0.9)	1.6 (1.1)	0.14	0.58
Sexual orientation is an important characteristic of people.	3.8 (1.1)	3.4 (1.3)	−0.21	0.40
Knowing a person's sexual orientation tells you a lot about them.	2.4 (1.4)	2.6 (1.4)	0.15	0.57
It's useful to group people according to their sexual orientation.	1.6 (0.8)	1.6 (0.7)	−0.08	0.75
Lesbian, gay, and bisexual people are known to be promiscuous and tend to be in non-monogamous relationships.	1.6 (0.8)	1.4 (0.6)	−0.20	0.43
Lesbian, gay, and bisexual partnerships are based on convenience rather than commitment.	1.4 (0.7)	1.3 (0.4)	−0.29	0.27
Mean (of 18 items)	1.8 (0.5)	1.8 (0.5)	−0.17	0.51

Note. Two items reverse-coded for Beliefs Towards Lesbian, Gay, and Bisexual People (4 & 6). Scale is on a 1 to 5 rating with higher indicating more negative beliefs.

**Table 5:** Beliefs Towards Transgender People (17 items).

Beliefs towards Transgender People	Pre M (SD)	Post M (SD)	d	p-val
Men who cross-dress for sexual pleasure disgust me	1.2 (0.4)	1.3 (0.4)	0	>0.99
Sex change operations are morally wrong	1.4 (0.6)	1.3 (0.4)	−0.25	0.33
God made two sexes and two sexes only	1.5 (0.8)	1.3 (0.4)	−0.20	0.43
If a friend wanted to have his penis removed in order to become a woman, I would openly support him	1.6 (0.9)	1.8 (1.2)	0.21	0.42
A man who dresses as a woman is a pervert	1.2 (0.4)	1.2 (0.4)	0	>0.99
It is morally wrong for a woman to present herself as a man in public	1.2 (0.4)	1.2 (0.4)	0	>0.99
Women who see themselves as men are abnormal	1.4 (0.7)	1.2 (0.4)	−0.25	0.33
People are either men or women	1.6 (1.1)	1.3 (0.6)	−0.17	0.51
Feminine men make me feel uncomfortable	1.2 (0.4)	1.3 (0.4)	0	>0.99
Feminine boys should be cured of their problem	1.2 (0.4)	1.2 (0.4)	0	>0.99
I would avoid talking to a woman if I knew she had surgically created a penis and testicles	1.1 (0.3)	1.2 (0.4)	0	>0.99
If I found out that my best friend was changing their sex, I would freak out	1.4 (0.6)	1.4 (0.6)	0.14	0.58
If I saw a man on the street that I thought was really a woman, I would ask him if he was a man or a woman	1.5 (0.9)	1.3 (0.6)	−0.25	0.33
I have behaved violently towards a woman because she was too masculine	1.2 (0.4)	1.2 (0.4)	0	>0.99
If I encountered a male who wore high-heeled shoes, stockings and makeup, I would consider beating him up	1.2 (0.4)	1.2 (0.4)	0	>0.99
Same-sex marriage is unnatural and has no sanctity.	1.2 (0.4)	1.2 (0.4)	0	>0.99
Same-sex marriage should not be a legal right.	1.2 (0.4)	1.2 (0.4)	0	>0.99
Mean (of 17 items)	1.3 (0.4)	1.3 (0.4)	−0.12	0.63

Note. One item reverse-coded for Beliefs Towards Trans scale (4) and two items for Beliefs Towards LGB (4 & 6). Scales is on a 1 to 5 rating with higher indicating more negative beliefs.

### 3.6 Self-efficacy

Table 6 shows that self-efficacy improved significantly from pre- to post-training for all 9 items (total score, d = 1.32) with effect sizes ranging from d = 0.56 to d = 1.46.

**Table 6:** Self-efficacy (9 items).

I am confident in…	Pre M (SD)	Post M (SD)	d	p-value
my knowledge of the terminology (sexual orientation, sex at birth, gender identity, preferred pronouns, families of choice) commonly used in the LGBTQ+ discourse.	3.2 (0.9)	4.2 (0.4)	1.22	<0.01
my ability to describe the health care disparities experienced by LGBTQ+ populations.	2.9 (0.9)	4.2 (0.4)	1.46	<0.01
demonstrating understanding of communication skills.	3.5 (0.8)	4.3 (0.5)	0.75	0.01
my ability to describe MSK resources for LGBTQ+ patients.	2.5 (0.9)	3.8 (0.8)	1.31	<0.01
my skills to create a safe and welcoming environment for LGBTQ+ patients.	3.7 (0.9)	4.3 (0.6)	0.64	0.02
my ability to describe the challenges of engaging and supporting the family of choice around pivotal cancer care decisions.	3.4 (0.9)	4.3 (0.5)	0.88	<0.01
my ability to provide a safe and welcoming environment for patients to disclose and discuss their LGBTQ+ status.	3.9 (0.8)	4.4 (0.5)	0.56	0.04
my ability to empathically enquire about LGBTQ+ patients' support system and invite their families of choice in medical decision making.	3.6 (0.9)	4.4 (0.5)	0.83	<0.01
my ability to have respectful conversations about who the decision-makers and support people are for my LGBTQ+ patients.	3.8 (1.0)	4.4 (0.5)	0.61	0.03
Total Score	3.4 (0.6)	4.3 (0.4)	1.32	<0.01

## 4 Discussion

This integrated training aimed to enhance the communication skills of multidisciplinary oncology HCPs when caring for LGBTQ+ patients with cancer and their families. Results showed the training was feasible, acceptable to participants, and led to improvements in LGBTQ+ healthcare knowledge and self-efficacy from pre- to post-training, consistent with prior preliminary evaluations [24], [25]. Given that many HCPs report low confidence in caring for LGBTQ+ patients, inadequate LGBTQ+ health knowledge, and an explicit desire for additional communication training [12], [13], [14], [15], the increase in participants' self-efficacy scores across all measured items is particularly relevant. This training complements other initiatives that seek to mitigate LGBTQ+ health inequities in the cancer context [18], [19], [21], [30], [31] and provides participants with core communication competencies needed to ensure person- and family-centered care delivery.

Future steps will include scaling this training with controlled testing of a larger sample, as well as the development and evaluation of additional modules focused on LGBTQ+ inclusive communication in end-of-life and bereavement settings. Specifically, we will aim to examine this comprehensive training among physicians and advanced practice providers (i.e., physician assistants, nurse practitioners) working in a range of practice settings (e.g., ambulatory, inpatient) to bolster HCP knowledge, attitudes, and confidence in providing person-centered communication for LGBTQ+ patients and their families across the cancer continuum. As this training is scaled, we also aim to gather patient- and family-reported outcomes (e.g., perceived HCP empathy [32], feeling heard and understood [33], [34], [35]). We anticipate this training will have clinical utility for HCPs in diverse practice specialties and disciplines to improve cancer care quality.

### 4.1 Limitations

The small sample of participants based at a single comprehensive cancer center limits the generalizability of our findings. Participants' high baseline knowledge of LGBTQ+ health in conjunction with their propensity to be more interested in or comfortable with LGBTQ+ related communication skills reflects a ceiling effect and potential self-selection bias that likely limited our ability to measure significant changes in knowledge and attitudes from pre- to post-training, as well as a need to recruit HCPs earlier in their education and careers during future evaluation. While some participants had previously attended a Comskil training, we did not assess differences based on attendance history. The use of standardized patients, while beneficial for preliminary training assessment, does not replace the need to investigate the transfer of skills to clinic settings and patient outcomes related to training effectiveness in the future.

### 4.2 Innovation

This training provides a novel approach to promoting safe, respectful communication for LGBTQ+ patients and their families, filling substantial research, education, and practice gaps. Extant trainings that aim to improve LGBTQ+ sensitivity and cultural humility focus on didactic knowledge-based content, case-based discussions, and interactive exercises (e.g., debriefing about a role play or HCP-patient encounter) [19], [21]. However, the Comskil model is a longstanding approach to healthcare communication that empowers participants to practice didactic strategies and skills through experiential learning in role plays with real-time feedback for participants to strengthen communication approaches [26], [36], [37]. Thus, the current training provides an innovative opportunity for participants to apply population-focused data in a practice-oriented learning environment that not only seeks to enhance knowledge but also attitudes and self-efficacy.

## 5 Conclusion

Our findings support the feasibility, acceptability, and preliminary efficacy of an integrated LGBTQ+ inclusive communication training for multidisciplinary oncology HCPs. Results highlight the connection between communication training and improved HCP self-efficacy in communicating with LGBTQ+ people. Despite pervasive inadequate and disrespectful care towards LGBTQ+ people [1], [2], [3], [4], [5], [6], [7], [8], [38], [39], trainings to improve HCP communication remain scarce [18], [21], [30]. This training fills a crucial gap in LGBTQ+ inclusive cancer care by providing well-established strategies and increasing HCP communication confidence through experiential learning [26], [36], [37].

## CRediT authorship contribution statement

**William E. Rosa:** Writing – review & editing, Writing – original draft, Investigation, Funding acquisition, Data curation, Conceptualization. **Smita C. Banerjee:** Writing – review & editing, Supervision, Investigation, Conceptualization. **Meghan McDarby:** Writing – review & editing, Investigation. **Amanda Kastrinos:** Writing – review & editing, Investigation. **Elizabeth Schofield:** Writing – review & editing, Formal analysis, Data curation. **Kimberly D. Acquaviva:** Writing – review & editing. **Koshy Alexander:** Writing – review & editing. **Patricia A. Parker:** Writing – review & editing, Supervision, Investigation, Conceptualization.

## Funding

This research is supported by the 10.13039/100015348Cambia Health Foundation Sojourns Scholars Leadership Award and 10.13039/100000867Robert Wood Johnson Foundation Harold Amos Medical Faculty Development Program (awarded to WER). WER, SCB, MM, AK, KA, and PAP acknowledge the NIH/National Cancer Institute comprehensive cancer center award P30 CA008748. AK is supported by supported by NIH/NCI Award Number K99CA27965. The authors have no other disclosures.

## Declaration of competing interest

The authors have no disclosures.
